# Improving diagnostic accuracy of routine EEG for epilepsy using deep learning

**DOI:** 10.1093/braincomms/fcaf319

**Published:** 2025-08-25

**Authors:** Émile Lemoine, Denahin Toffa, An Qi Xu, Jean-Daniel Tessier, Mezen Jemel, Frédéric Lesage, Dang Khoa Nguyen, Elie Bou Assi

**Affiliations:** Department of Neuroscience, Université de Montréal, Montréal, Canada, H3T 1J4; Institute of Biomedical Engineering, Polytechnique Montréal, Montréal, Canada, H3T 0A3; Neuroscience Axis, Centre de Recherche du Centre Hospitalier de l’Université de Montréal (CRCHUM), Montréal, Canada, H2X 0A9; Department of Neuroscience, Université de Montréal, Montréal, Canada, H3T 1J4; Neuroscience Axis, Centre de Recherche du Centre Hospitalier de l’Université de Montréal (CRCHUM), Montréal, Canada, H2X 0A9; Neuroscience Axis, Centre de Recherche du Centre Hospitalier de l’Université de Montréal (CRCHUM), Montréal, Canada, H2X 0A9; Department of Neuroscience, Université de Montréal, Montréal, Canada, H3T 1J4; Neuroscience Axis, Centre de Recherche du Centre Hospitalier de l’Université de Montréal (CRCHUM), Montréal, Canada, H2X 0A9; Department of Neuroscience, Université de Montréal, Montréal, Canada, H3T 1J4; Neuroscience Axis, Centre de Recherche du Centre Hospitalier de l’Université de Montréal (CRCHUM), Montréal, Canada, H2X 0A9; Institute of Biomedical Engineering, Polytechnique Montréal, Montréal, Canada, H3T 0A3; Department of Neuroscience, Université de Montréal, Montréal, Canada, H3T 1J4; Neuroscience Axis, Centre de Recherche du Centre Hospitalier de l’Université de Montréal (CRCHUM), Montréal, Canada, H2X 0A9; Department of Neuroscience, Université de Montréal, Montréal, Canada, H3T 1J4; Neuroscience Axis, Centre de Recherche du Centre Hospitalier de l’Université de Montréal (CRCHUM), Montréal, Canada, H2X 0A9

**Keywords:** epilepsy, EEG, deep learning, diagnosis, computer-assisted

## Abstract

The yield of routine EEG to diagnose epilepsy is limited by low sensitivity and the potential for misinterpretation of interictal epileptiform discharges. Our objective is to develop, train and validate a deep learning model that can identify epilepsy from routine EEG recordings, complementing traditional interpretation based on identifying interictal discharges. This is a retrospective cohort study of diagnostic accuracy. All consecutive patients undergoing routine EEG at our tertiary care centre between January 2018 and September 2019 were included. EEGs recorded between July 2019 and September 2019 constituted a temporally shifted testing cohort. The diagnosis of epilepsy was established by the treating neurologist at the end of the available follow-up period, based on clinical file review. Original EEG reports were reviewed for IEDs. We developed seven novel deep learning models based on Vision Transformers and Convolutional Neural Networks, training them to classify raw EEG recordings. We compared their performance to interictal discharge-based interpretation and two previously proposed machine learning methods. The study included 948 EEGs from 846 patients (820 EEGs/728 patients in training/validation, 128 EEGs/118 patients in testing). Median follow-up was 2.2 years and 1.7 years in each cohort, respectively. Our flagship Vision Transformer model, DeepEpilepsy, achieved an area under the receiver operating characteristic curve of 0.76 (95% confidence interval: 0.69–0.83), outperforming interictal discharge-based interpretation (0.69; 0.64–0.73) and previous methods. Combining DeepEpilepsy with interictal discharges increased the performance to 0.83 (0.77–0.89). DeepEpilepsy can identify epilepsy on routine EEG independently of interictal discharges, suggesting that deep learning can detect novel EEG patterns relevant to epilepsy diagnosis. Further research is needed to understand the exact nature of these patterns and evaluate the clinical impact of this increased diagnostic yield in specific settings.

## Teaser

Lemoine *et al*. report that deep learning can improve the diagnostic yield of routine EEG for epilepsy. Their model, DeepEpilepsy, outperformed traditional interpretation based on epileptiform discharges and enhanced diagnostic accuracy when combined with conventional EEG analysis.

## Graphical Abstract

**Graphical Abstract:**
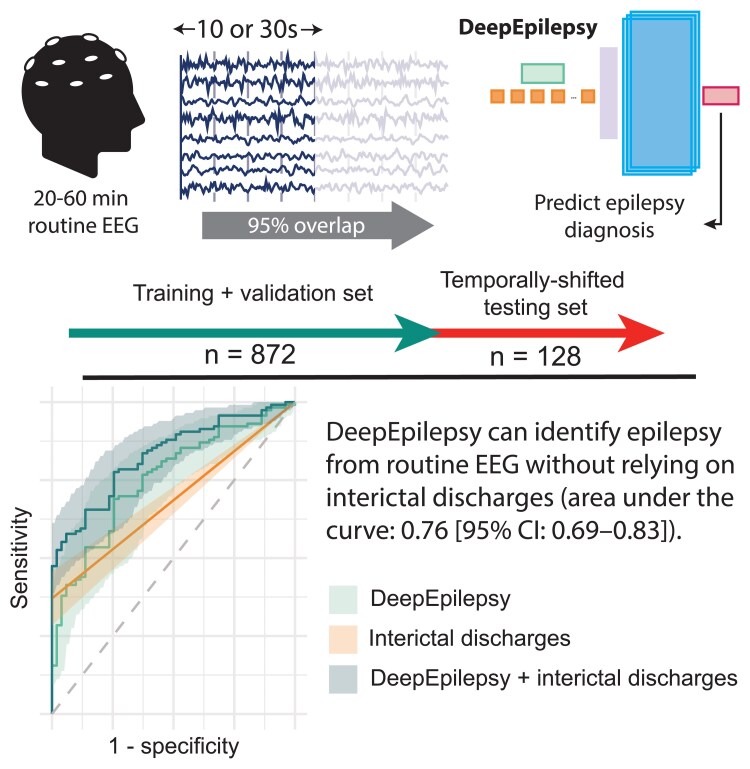

## Introduction

The diagnosis of epilepsy is notoriously challenging. It relies on the occurrence of either two seizures more than 24 h apart, one seizure and a high risk of another, or the presence of an epilepsy syndrome.^1^ Despite this clear definition, the rate of misdiagnosis remains high,^2,3^ being highly dependent on the ability to collect a clear clinical history and accurately interpret the electroencephalogram (EEG).

The EEG can capture ictal and interictal activity, namely interictal epileptiform discharges (IEDs), which are highly specific for epilepsy (98%).^4^ A scalp EEG is cost-effective and technically straightforward, with standard acquisition protocols that have been put in place by the International League Against Epilepsy.^5,6^ However, the sensitivity of a single routine EEG for IEDs is 20–50%, and only 17% in adults after a first unprovoked seizure.^7-9^ Furthermore, the interrater reliability for IEDs is fair to moderate even among experts, with a kappa of 35–50%.^10-12^ Consequently, the EEG has limitations as a diagnostic tool in patients with suspected seizures, with EEG misinterpretation contributing to diagnostic errors in epilepsy.^13^ The identification of additional biomarkers beyond IEDs could help overcome these limitations and improve diagnostic accuracy.^14,15^

In recent decades, efforts have focused on overcoming the limitations of traditional EEG interpretation by identifying alternative epilepsy biomarkers through computational methods.^16-20^ While these approaches have shown promise, their translation to clinical practice has been limited by several factors: modest performance,^16,21,22^ small^23^ or lack of dedicated^24-28^ testing set, exclusion of patients with neurological co-morbidities or abnormal EEGs,^23-25^ and reliance on IED detection.^24,25^ As a result, the expected diagnostic accuracy of these approaches in a real-world population is uncertain.

Deep learning (DL) has emerged as a powerful tool for the analysis of complex signals. DL models can autonomously extract features from time-series or images by optimizing millions of parameters on large datasets. DL has been applied to EEG to decode brain signals for brain-computer interface,^29^ predict delirium^30^ and automatically detect IEDs.^31,32^ Given DL’s capacity to capture the complex brain dynamics, we hypothesized that it could enhance the detection of epilepsy-specific patterns on routine EEG recordings.

The present study seeks to address these questions: can modern DL models detect epilepsy on interictal EEG, even in the absence of IEDs? What are the potential diagnostic performances of a DL-assisted EEG interpretation for epilepsy? And what sample size is required to train such models?

## Materials and methods

### Study design

This is a retrospective study on a consecutive cohort of patients undergoing routine EEG in a single tertiary care centre in Montreal, Canada.

### Participants

We included all patients who underwent a routine EEG (20–60-min, with or without sleep deprivation) between January 2018 and September 2019 at the Centre Hospitalier de l’Université de Montréal (CHUM). Exclusion criteria were the absence of follow-up after the EEG, an uncertain diagnosis of epilepsy at the end of the available follow-up period, or an EEG performed in a hospitalized patient. Under a pre-specified protocol, one neurology resident (É.L.) and three students (A.Q.X., M.J., J.-D.T.) collected data from the electronic health record for each visit, including baseline characteristics (age, sex), co-morbidities, number of antiseizure medications and presence of a focal lesion on neuroimaging. They also reviewed the EEG report for the presence of IED(s) and abnormal background slowing. All clinical information was stored on a REDCap database hosted on the CHUM research centre’s servers.

We separated the cohort into two independent subsets according to the date of the EEG. Recordings before 15 July 2019, comprised the training and validation set, while recordings after 15 July 2019, comprised the testing set. We excluded from the testing set any recording from a patient already included in the training and validation set. The training and validation set was further separated into a training set and a validation set in a random fashion (80%/20% split).

### Test methods

#### Reference standard

The reference standard is the diagnosis of epilepsy according to the treating physician at the end of the available follow-up period. This diagnosis is based on the ILAE definition of epilepsy, i.e. having had two unprovoked seizures more than 24 h apart or one unprovoked seizure and be considered at high (>60%) risk of seizure recurrence, or being diagnosed with an epilepsy syndrome.^1^ The final diagnosis at the end of the follow-up period was used, as opposed to the speculated diagnosis at the time of the EEG, because the follow-up period provides additional information such as imaging, additional EEG recordings, video-monitoring admissions and seizure recurrences.

#### EEG recording

EEGs were recorded using a standardized protocol on a Nihon Kohden EEG system, following national recommendations.^33^ Awake EEGs, 20–30 min long, were recorded at 200 Hz with 21 electrodes arranged with the 10–20 system. They included two 90-s periods of hyperventilation (except in patients > 80 years old, uncooperative, or with medical contraindications) and photic stimulation from 4 to 22 Hz. Patients were also instructed to open or close their eyes at several times. Sleep deprived recordings lasted 60 min, with the same activation procedures. Technologists annotated the EEG in real-time. For this study, EEGs were converted to an average referential montage (A1–A2), saved to EDF format, and stored on the CHUM research centre’s server for analysis.

#### Automated processing of EEG and classification

**Figure 1:**
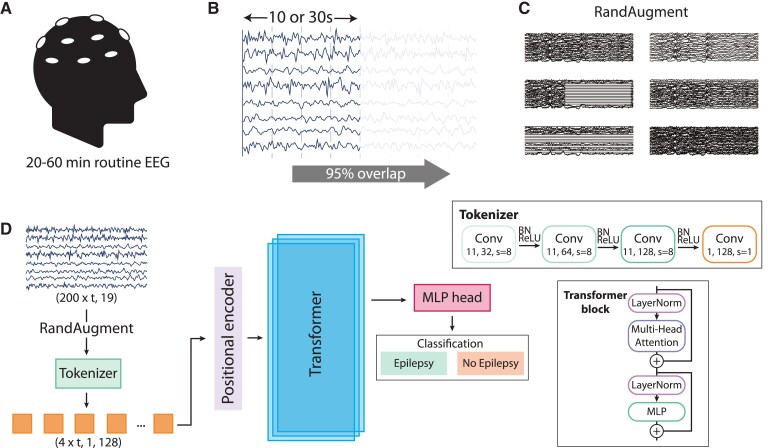
**Details of the DeepEpilepsy Transformer model.** (**A**) The input data consists of standard 20–60 min routine EEG recordings. (**B**) EEGs are segmented into 10- or 30-s windows with 95% overlap. (**C**) EEG windows are processed through RandAugm with 50% probability. (**D**) EEG windows are processed through a tokenizer (**D**, upper right: convolutional tokenizer) followed by positional encoding. The tokens are then input into a Transformer model with a MLP head for classification according to the diagnosis of epilepsy. Final predictions are derived from the median of all windows for a single EEG recording. BN, batch normalization; Conv, convolutional layer; MLP, multilayer perceptron; RandAugment, random augmentation; ReLU, rectified linear unit.

The index test is the classification of the EEG recordings using machine learning. We developed DeepEpilepsy, a Vision Transformer (ViT) model that takes raw EEG segments as input and outputs a probability of the diagnosis of epilepsy (Fig. 1). EEGs in average referential montage (19 channels) were segmented into overlapping 10- or 30-s windows (95% overlap) and directly used as input into the DL models. The input dimensions were 19×200t, where *t* is the window size in seconds. We initially explored two window sizes (10 and 30 s) based on common practice in EEG interpretation and computational constraints.^17^ A 95% overlap between segments was used to provide sufficient data augmentation while maintaining computational feasibility. We also investigated the impact of other window sizes on performances (Supplementary Table 6). The model configurations for the ViT models, including DeepEpilepsy, are presented in Supplementary Table 1. To enhance model generalization, we applied a random data augmentation algorithm during training.^34^ For each segment, an augmentation was drawn randomly from a set of transformations, which included filtering (band-pass, low-pass, high-pass), masking (channel, time) and adding noise (Supplementary Fig. 1). These were applied with a 50% probability and randomized intensity. We performed a Bayesian hyperparameter search on the training and validation set to choose DeepEpilepsy’s final configuration. We also investigated different learning rates, weight decay and batch size values. The final models were trained on the entire training and validation set. The optimization hyperparameters and model specifications are described in Supplementary Table 4.

In addition, we implemented other Deep Learning models (ViT and ConvNeXt; Supplementary Tables 1 and 2), as well as two previously described methods: the ShallowConvNet inspired by the ‘Filter Bank Common Spatial Patterns’ algorithm (Supplementary Table 3),^35^ and a feature-extraction framework relying on the extraction of linear and nonlinear EEG markers that are used as input into a classifier (LightGBM).^21^ These methods are described in details in Supplementary Methods 1.

To obtain the diagnostic performances, the final models/procedures were applied to the testing set. This resulted in a single predicted probability for each EEG segments. To obtain one prediction per EEG recording, we aggregated the predicted probabilities at the EEG-level using the median of the predicted values. In cases where patients had multiple EEGs, each recording was treated as an independent observation. A sensitivity analysis excluding repeated EEGs was performed to assess potential bias.

We further evaluated DeepEpilepsy in a specific subgroup of patients that were not yet diagnosed with epilepsy at the time of the index EEG (i.e. undergoing evaluation for suspected seizures). We also measured the performance bias across different subgroups: age groups (18–40, 40–60 and >60 years old), sex, presence of focal lesion, presence of IED (absence, presence and uncertain), presence of slowing, sleep deprivation before EEG and number of ASM (0, 1, ≥2).

### Analysis

We calculated the area under the receiver operating characteristic curve (AUROC) using the probabilistic predictions for each model, with 95% confidence intervals estimated using DeLong’s method (single prediction by patient).^36^ We also computed the area under the precision-recall curve (AUPRC) with 95% confidence intervals estimated using bootstrap resampling (1000 iterations). For comparison, we tested the classification performance of IEDs alone (presence versus absence). We also tested a two-step classification using IEDs first (traditional EEG interpretation), followed by DeepEpilepsy if IEDs were absent (DL interpretation).

The optimal classification threshold was obtained using the validation cohort, minimizing the distance between the curve and the upper left corner of the ROC graph. This threshold was then applied to compute sensitivity, specificity, negative predictive value and positive predictive value on the testing set.

We performed additional analyses to better quantify the effect of window duration and random augmentation on DeepEpilepsy. For segment duration, we re-trained the model using window durations of s, 10, 15, 30, 45 and 60 s (with fixed 1.5 s overlap to maintain consistent training sample size). For random augmentation, we re-trained DeepEpilepsy eight times with and without RandAugment (20 epochs each). Performances between augmented and non-augmented models were compared with AUROC on the testing set as the performance metric.

We performed an exploratory analysis of the embeddings learned by DeepEpilepsy and ShallowConvNet to better understand the patterns captured by both models (Supplementary Methods 2). Embeddings are the internal representations that deep learning models create while processing raw EEG data—they represent how the model ‘sees’ the EEG after transforming it through multiple layers, without any pre-specified features. These learned representations differ from traditional EEG features and can provide insights into what patterns the model considers important for classification.

### Sample size

Using Obuchowski’s method,^37^ with a 60% epilepsy prevalence, a power of 0.9 and a significance level of 0.0071 (adjusted from 0.05 divided by 7 DL models), a minimum of 126 EEGs is required to detect an AUROC of 0.70.

### Standard protocol approvals, registrations and patient consents

Ethics approval was granted by the CHUM Research Centre’s Research Ethics Board (REB) (Montreal, Canada, project number: 19.334). The REB waived informed consent due to the lack of diagnostic/therapeutic intervention and minimal risk to participants. All methods followed Canada’s Tri-Council Policy statement on Ethical Conduct for Research Involving Humans.

## Results

### Participants

**Figure 2:**
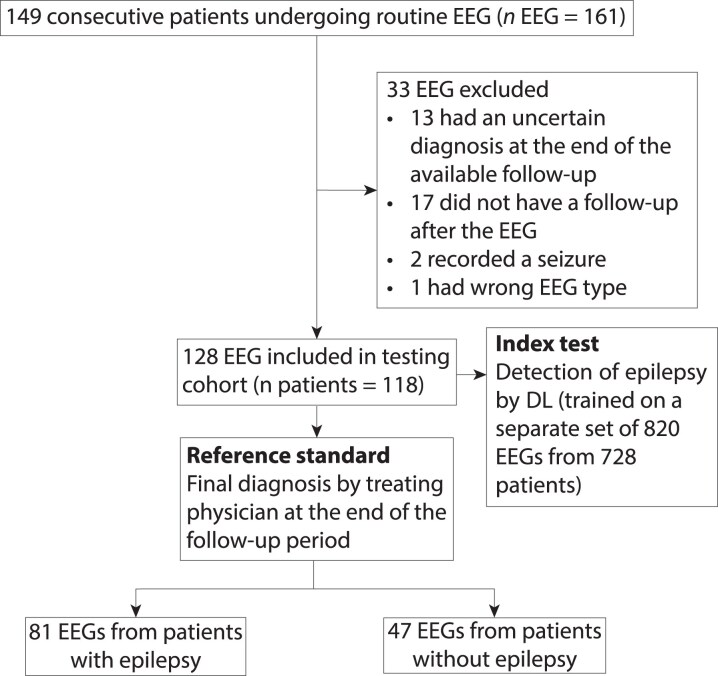
Flowchart of patients included in the testing cohort.

After exclusion, 948 EEGs from 846 patients were included: 820 EEGs in the training/validation set (728 patients) and 128 EEGs in the testing set (118 patients), with no patient overlap (Table 1). Before exclusion, 1185 EEGs from 1 067 patients and 161 EEGs from 149 patients met the inclusion criteria for the training and testing cohorts, respectively. Reasons for exclusion were absence of follow-up after the EEG, uncertain diagnosis at the end of available follow-up, seizure during the EEG and wrong EEG type (i.e. performed in a hospitalized patient) (Fig. 2). Median age were 49 and 51.5 (IQR: 32–62 and 30–62.5) and the proportion of female were 51% and 62.5% in the training and testing cohorts, respectively. Median follow-up was 2.2 years (IQR: 1.0–2.9) and 1.7 years (IQR: 0.9–2.3). Epilepsy prevalence was 63% in both sets.

**Table 1:** Description of the training (EEG recordings between January 2018 and July 2019) and testing cohorts (EEG recordings between July and September 2019)

	Training/validation cohort (*n* = 820)		Testing cohort (*n* = 128)	
	Epilepsy	No epilepsy	Epilepsy	No epilepsy
Number of EEGs	517	303	81	47
Sex = female (%)	259 (50.1)	159 (52.5)	54 (66.7)	26 (55.3)
Age (median [IQR])	42.00 [29.00, 58.00]	57.00 [41.00, 67.00]	37.00 [25.00, 57.00]	60.00 [50.50, 71.00]
Total follow-up after EEG in weeks (median [IQR])	133.50 [95.75, 173.00]	59.00 [17.00, 116.00]	99.50 [70.25, 125.00]	62.00 [17.00, 102.00]
Epilepsy type (%)				
Focal	370 (71.6)		49 (60.5)	
Generalized	119 (23.0)		26 (32.1)	
Unknown	28 (5.4)		6 (7.4)	
Age of epilepsy onset (median [IQR])	22.00 [13.00, 40.00]		23.00 [14.00, 48.00]	
Seizure recurrence after EEG (%)	269 (52.0)	0 (0.0)	44 (54.3)	0 (0.0)
Number of days since last seizure (median [IQR])	237 [56, 1134]		118 [44, 467]	
Number of epilepsy risk factors (median [IQR])	3 [1, 4]	2 [1, 4]	2 [1, 3]	1 [0, 3]
History of epilepsy surgery (%)	60 (11.6)	0 (0.0)	4 (4.9)	0 (0.0)
Number of ASM (%)				
0	55 (10.6)	253 (83.5)	17 (21.0)	42 (89.4)
1	280 (54.2)	36 (11.9)	34 (42.0)	5 (10.6)
2	123 (23.8)	12 (4.0)	19 (23.5)	0 (0.0)
3	47 (9.1)	2 (0.7)	6 (7.4)	0 (0.0)
4	10 (1.9)	0 (0.0)	5 (6.2)	0 (0.0)
5	2 (0.4)	0 (0.0)	0 (0.0)	0 (0.0)
Focal lesion on brain imaging (%)	223 (43.1)	84 (27.7)	31 (38.3)	10 (21.3)
Sleep deprived EEG (%)	62 (12.0)	50 (16.5)	22 (27.2)	8 (17.0)
IED (%)				
Absence	333 (64.4)	282 (93.1)	42 (51.9)	46 (97.9)
Presence	139 (26.9)	2 (0.7)	30 (37.0)	0 (0.0)
Uncertain	45 (8.7)	19 (6.3)	9 (11.1)	1 (2.1)
Abnormal slowing on EEG (%)	199 (38.5)	46 (15.2)	32 (39.5)	10 (21.3)

In the training cohort, 141 EEGs (17%) showed definite IEDs and 64 (8%) showed uncertain IEDs. Two definite IEDs were found in patients without epilepsy. In the testing cohort, 30 EEGs (23%) showed definite IEDs and 10 (8%) showed uncertain IEDs, with all definite IEDs in patients with epilepsy.

### Test results

**Figure 3:**
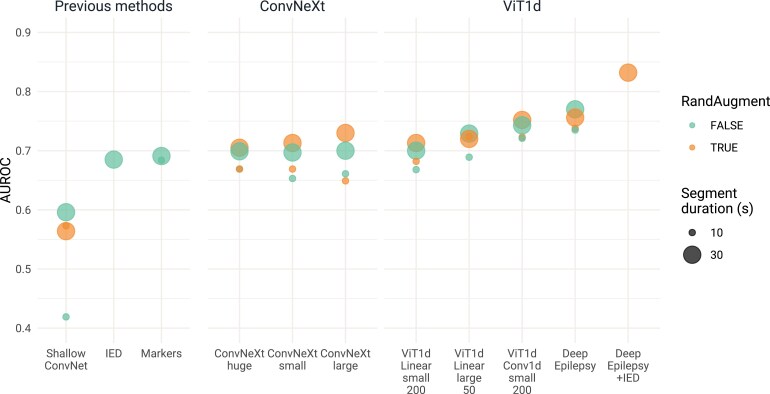
**Diagnostic performances of automated EEG analysis for the diagnosis of epilepsy on the testing set (*n* = 128).** Our flagship model, DeepEpilepsy, is shown alone and combined with traditional EEG interpretation based on the identification of IED. The other novel approaches shown are ViTs and ConvNeXt using different configurations (size: small, large, huge; tokenizers: convolutional or linear; window size: 50 pt or 200 pt) as well as presence of RandAugm and the duration of EEG segments used as input. Previous methods are the ShallowConvNet,^23^ extraction of computational markers^21^ and the presence of IEDs on EEG. AUROC, area under the receiver operating characteristic curve; IED, interictal epileptiform discharges; ViT, Vision Transformers.

**Figure 4:**
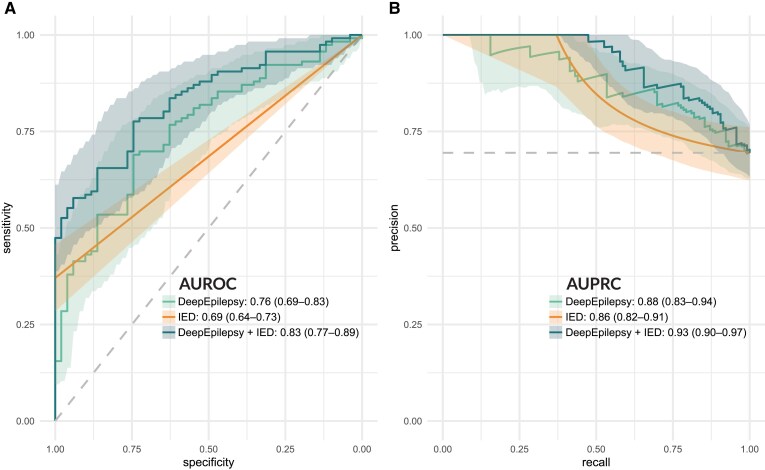
**Diagnostic performances on the testing set (*n* = 128).** (**A**) ROC curves for DeepEpilepsy, IEDs only and DeepEpilepsy combined with IEDs in the testing cohort. (**B**) Precision-recall curves for the three approaches. AUPRC, area under the precision-recall curve; AUROC: area under the receiver operating characteristic curve; IED, interictal epileptiform discharges.

The AUROC for the diagnosis of epilepsy in the testing cohort for every approach is pictured in Fig. 3. For DeepEpilepsy, the AUROC was 0.76 (95% CI: 0.69–0.83) and AUPRC of 0.88 (0.83–0.94) (Fig. 4). Using the threshold computed on the validation cohort (0.86), there were 75 true positives, 38 true negatives, 13 false positive and 41 false negatives, equating to a sensitivity of 64.7%, a specificity of 74.5%, a positive predicted value (PPV) of 85.2% and a negative predictive value (NPV) of 48.1%. For comparison, when using the presence of IEDs on EEG (as per the EEG report) as the index test, the sensitivity is 37.0%, specificity is 100.0%, PPV is 100.0% and NPV is 41.1%, with an AUROC of 0.69 (95% CI: 0.64–0.73) and AUPRC of 0.86 (0.82–0.91) (Fig. 4). The AUROC of DeepEpilepsy was higher than any other method, although this was only statistically significant when compared to the ShallowConvNet models (AUROC: 0.60, 95% CI: 0.50–0.69). The diagnostic performances of all methods are shown in Table 2.

**Table 2:** Classification performances on the testing set for all machine learning methods

	Segment duration (s)	RandAugment	AUC
DeepEpilepsy	30	False	0.77 (0.69–0.84)
DeepEpilepsy	30	True	0.76 (0.68–0.83)
ViT1d, Conv tokenizer, small	30	True	0.75 (0.68–0.83)
ViT1d, Conv tokenizer, small	30	False	0.74 (0.66–0.82)
DeepEpilepsy	10	True	0.74 (0.66–0.81)
DeepEpilepsy	10	False	0.73 (0.64–0.81)
ConvNeXt, large	30	True	0.73 (0.65–0.81)
ViT1d, Linear tokenizer, large	30	False	0.73 (0.65–0.80)
ViT1d, Conv tokenizer, small	10	True	0.72 (0.64–0.80)
ViT1d, Linear tokenizer, large	10	True	0.72 (0.64–0.80)
ViT1d, Linear tokenizer, large	30	True	0.72 (0.64–0.80)
ViT1d, Conv tokenizer, small	10	False	0.72 (0.64–0.80)
ConvNeXt, small	30	True	0.71 (0.63–0.80)
ViT1d, Linear tokenizer, small	30	True	0.71 (0.63–0.79)
ConvNeXt, huge	30	True	0.71 (0.62–0.79)
ConvNeXt, huge	30	False	0.70 (0.61–0.78)
ConvNeXt, large	30	False	0.70 (0.62–0.78)
ViT1d, Linear tokenizer, small	30	False	0.70 (0.61–0.78)
ConvNeXt, small	30	False	0.70 (0.61–0.78)
Feature extraction with LightGBM	30		0.69 (0.60–0.78)
ViT1d, Linear tokenizer, large	10	False	0.69 (0.60–0.76)
Feature extraction with LightGBM	10		0.68 (0.59–0.77)
ViT1d, Linear tokenizer, small	10	True	0.68 (0.59–0.76)
ConvNeXt, huge	10	False	0.67 (0.58–0.76)
ConvNeXt, huge	10	True	0.67 (0.58–0.75)
ViT1d, Linear tokenizer, small	10	False	0.67 (0.58–0.75)
ConvNeXt, small	10	True	0.67 (0.58–0.76)
ConvNeXt, large	10	False	0.66 (0.58–0.75)
ConvNeXt, small	10	False	0.65 (0.57–0.74)
ConvNeXt, large	10	True	0.65 (0.56–0.73)
ShallowConvNet	30	False	0.60 (0.49–0.69)
ShallowConvNet	10	True	0.57 (0.47–0.67)
ShallowConvNet	30	True	0.56 (0.46–0.66)
ShallowConvNet	10	False	0.42 (0.32–0.51)

When using the two-step model as the index test (1: presence of IED classified as epilepsy, 2: if no IED: DeepEpilepsy models prediction), the AUROC was 0.83 (95% CI: 0.77–0.89) and AUPRC was 0.93 (0.90–0.96) (Fig. 4). The sensitivity, specificity, PPV and NPV were 73.2%, 74.5%, 86.7% and 55.1%.

A sensitivity analysis excluding repeated EEGs from the testing set showed similar performance, with DeepEpilepsy achieving an AUROC of 0.74 (*n* = 118; 95% CI: 0.65–0.81).

#### Subgroup analyses

In the testing cohort, 75 patients (64%) had an uncertain diagnosis at the time of the EEG, 28 of which were eventually diagnosed with epilepsy. In the 47 others, the most common final diagnoses were syncope/faintness (11), dementia-related fluctuations (6) and non-specific sensitive symptoms (5). Within this subgroup of uncertain diagnoses, 10 patients who were diagnosed with epilepsy showed IEDs and 6 had uncertain sharp transients (versus 1 in patients without epilepsy). The complete characteristics of the subgroup are detailed in Supplementary Table 5.

In the subgroup of 75 patients not diagnosed with epilepsy at the time of the EEG, DeepEpilepsy still had above-chance performances (AUROC: 0.69, 95% CI: 0.56–0.80), and the two-step model had the following performances: sensitivity of 65.6%, specificity of 76%, PPV of 63.6% and NPV of 77.6%, with an AUROC of 0.77 (0.65–0.87). The ROC curves for IEDs only, DeepEpilepsy and DeepEpilepsy combined with IEDs for this subgroup are shown in Supplementary Fig. 2.

**Figure 5:**
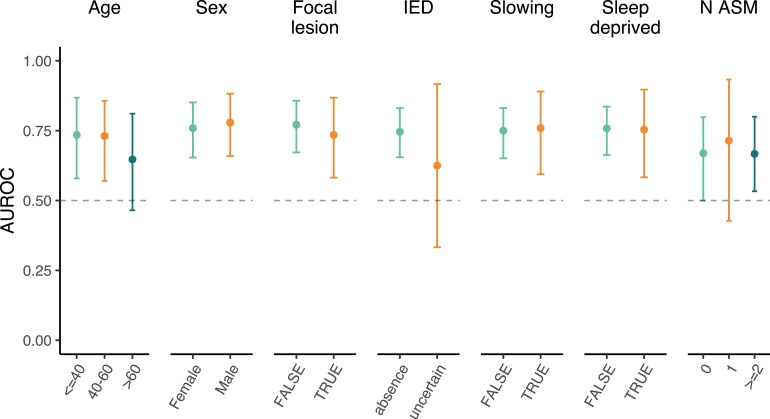
**Performance of DeepEpilepsy for classification of epilepsy diagnosis from routine EEG in different subgroups of the testing set.** The subgroups have the following sample sizes: (i) age < 40: *n* = 40, >40–≤60: *n* = 44, >60: *n* = 44; (ii) male: *n* = 48, female: *n* = 80; (iii) focal lesion: *n* = 41, no focal lesion: *n* = 87; (iv) uncertain IED: *n* = 10, absence of IED: *n* = 88. (v) focal slowing: *n* = 42, no focal slowing: *n* = 86; (vi) sleep deprived EEG: *n* = 30, awake EEG: *n* = 98; and (vii) no ASM: *n* = 59, one ASM: *n* = 39, ≥2 ASMs: *n* = 30. ASM, antiseizure medication; AUROC, area under the receiver operating characteristic curve; IED, interictal epileptiform discharges.

The results for other subgroups are presented in Fig. 5. Across all subgroups, performances were above chance except for patients > 60 years old and patients with a single antiseizure medication. Notably, in absence of IEDs (*n* = 98), AUROC was 0.74 (0.65–0.83), with NPV of 0.55%, PPV of 76%, sensitivity of 57% and specificity of 75%. By comparison, in patients where DeepEpilepsy predicted low epilepsy risk (*n* = 79), IEDs had a AUROC of 0.62 (0.56–0.68), with NPV of 55%, PPV of 100%, sensitivity of 24% and specificity of 100%. Also, DeepEpilepsy performed similarly in sleep deprived EEG and awake EEGs (AUROC = 0.76 [0.67–0.84] and 0.76 [0.58–0.90], respectively).

#### Sample size, segment duration and RandAugment analysis

**Figure 6:**
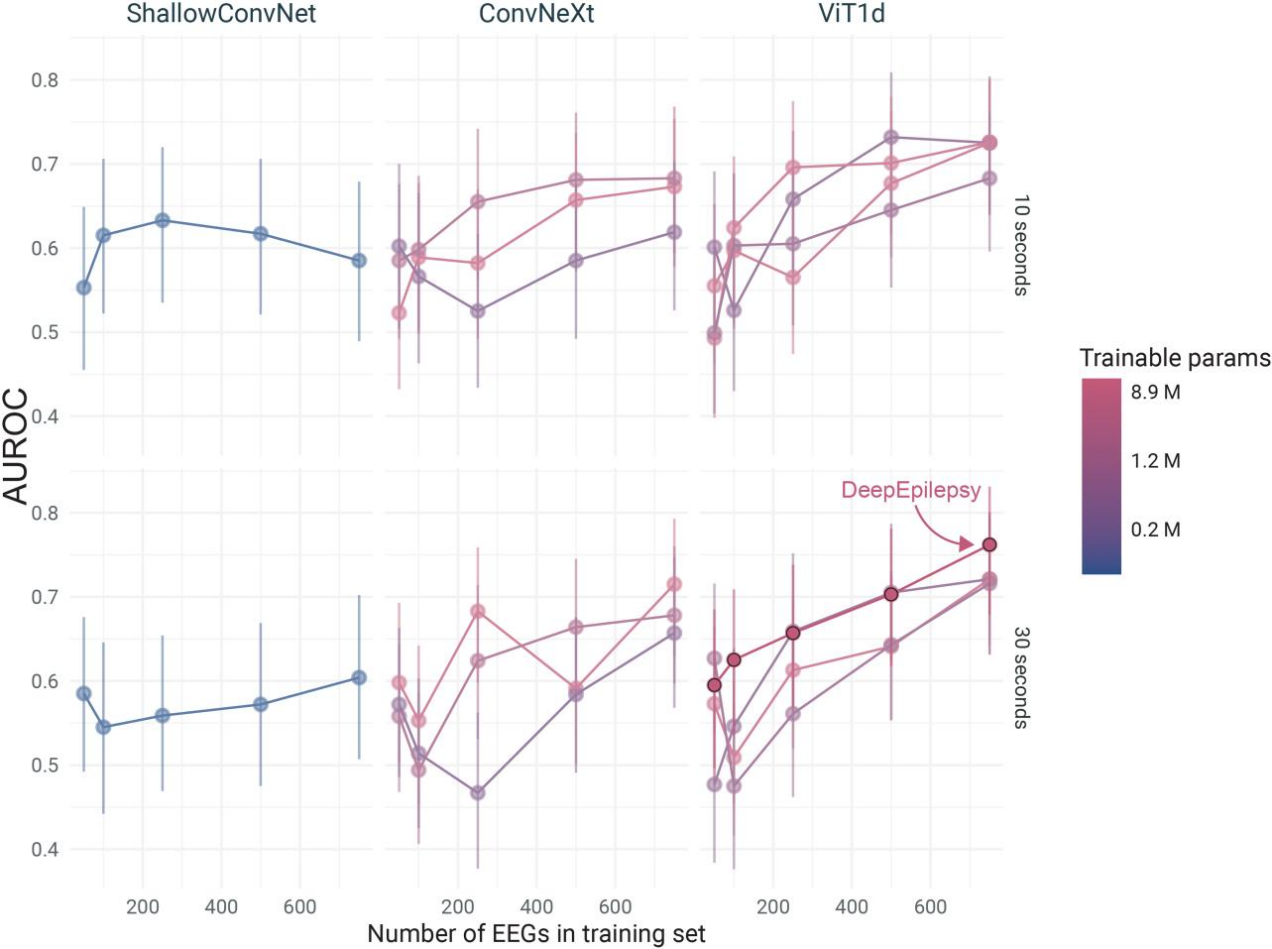
**Impact of training sample size on the performance of four deep learning models (ShallowConvNet, ConvNeXt, DeepEpilepsy and other Vision Transformers) for detecting epilepsy from EEG segments.** Performance is measured by the AUROC score on the testing set (*n* = 128), with models trained on varying numbers of EEGs (50, 100, 250, 500 and 750). The models were trained on 10 s (top row) and 30 s (bottom row) overlapping EEG segments. AUROC, area under the receiver operating characteristic curve; IED, interictal epileptiform discharges; ViT1d, Vision Transformer with one-dimension tokenizer.

We trained the different neural network models on subsets of the data (50, 100, 250, 500 and 750 EEGs) to assess the impact of the size of the training sample on performance (Fig. 6). With 10-s segments, the ShallowConvNet had highest performances when trained on 250 EEG recordings. The other models tended towards increased performances, with a ceiling at 500 EEGs. Using 30-s segments, the ShallowConvNet showed a slight increase in performances with increased training size, with a maximal AUROC of 0.6 at 750 EEGs. In contrast, the performance of the ConvNeXt and ViT models increased almost linearly with sample size, achieving the highest performances with 750 EEGs. In almost all cases, 500 EEGs was the minimal training size required to achieve above-chance performances. For reference, using our segmentation strategy, 500 EEGs resulted in 765 000 10-s overlapping segments or 500 000 30-s overlapping segments.

A systematic evaluation of segment durations showed that 30-s windows achieved marginally better performance than other window sizes, though the difference was not statistically significant (Supplementary Table 6). Models trained with RandAugment showed slightly higher peak performance but increased variability compared to models without data augmentation (max AUROC 0.73 versus 0.72, mean AUROC 0.71 versus 0.71, standard deviation of AUROC: 0.011 versus 0.017, *P* = 0.87; Supplementary Fig. 3).

#### Relationship between learned representations and traditional EEG features

Deep learning models such as DeepEpilepsy transform raw EEG signals into hidden representations (embeddings) that are optimal for distinguishing patients with and without epilepsy. To understand what patterns these models capture, we analysed how these embeddings relate with traditional EEG features (namely band power and entropy) using clustering analysis. For band power, DeepEpilepsy’s embeddings showed higher variance in the high-frequency range (>13 Hz), particularly in the 20–40, 40–75 and 75–100 Hz bands. In contrast, ShallowConvNet’s embeddings exhibited relatively higher variance in the low-frequency range (<10 Hz) (Supplementary Fig. 4). Although DeepEpilepsy showed significant heterogeneity across all frequency bands, ShallowConvNet had non-significant analysis of variance in the 20–40 Hz range (*P* = 0.24) Regarding entropy, both models showed significant heterogeneity across all frequencies, but ShallowConvNet displayed higher inter-cluster variance, especially for bands above 1.6 Hz, suggesting that this was a key feature learned by this model (Supplementary Fig. 5).

## Discussion

This study assessed the diagnostic performance of DL-based analysis of routine EEG for epilepsy. We developed and trained the DL models on 948 consecutive EEGs from 846 patients, testing them on a temporally shifted cohort of 128 EEGs from 118 patients. Our flagship model, DeepEpilepsy, had a testing AUROC of 0.76 (95% CI: 0.69–0.83), outperforming other methods including conventional IED-based interpretation and previously proposed computational methods. Combining the presence of IEDs with DL analysis increased the AUROC to 0.83 (95% CI: 0.77–0.89), demonstrating a potential for clinical translation.

Epilepsy diagnosis is primarily clinical, guided by individualized seizure recurrence risk assessment, which can be challenging due to limited reliable data.^1^ The identification of IEDs on rEEG is commonly used to support the diagnosis of epilepsy, but their low sensitivity and risk of over-interpretation can often lead to both over- or underdiagnosis.^13^ In our study, IEDs had an AUROC of 0.69 with a sensitivity as low as 37%. Our DL models provided higher overall diagnostic performances from the EEG than IEDs. Combining both approaches allowed to leverage the model’s higher sensitivity and the high specificity of IEDs. Currently, no definitive, quantitative, non-ictal biomarkers have been validated for clinical use.^1^ Although several studies have explored changes in the EEG such as shifts in band power^19,38,39^ or changes in entropy,^40,41^ many remain at the ‘proof-of-concept’ stage, limited by case-control designs or inadequate validation.^17^ More recent studies on computational analysis of EEG for the diagnosis of epilepsy have shown mixed results.^16,22^ Unlike prior work,^17^ our validation cohort corresponds to the group of patients in which the algorithm would be used in real-life, reducing bias in performance evaluation. While we chose a temporally shifted test set to mirror clinical implementation, future work could benefit from additional validation strategies such as nested cross-validation or multicentre testing. Furthermore, the gold-standard in our study was based on a thorough review of clinical notes with a median follow-up period of over two years, allowing the clinician to build a more complete clinical picture integrating seizure recurrence, imaging, video-EEG evaluations, or new clinical symptoms. This is in contrast with studies that based the diagnosis on the EEG report or a single clinical visit.^17^ These methodological strengths reduce bias and represent key steps towards the clinical integration of automated EEG analysis.^17^

DeepEpilepsy is based on the Transformer architecture,^42^ which has greatly advanced our capacity to model sequence data. Transformers have been adapted for EEG-based tasks such as eye-tracking,^43^ seizure prediction^44,45^ and decoding of motor patterns.^46^ A critical component in adapting Transformers to EEG is the tokenization method, which influences feature extraction and the timescales captured by the model. Previous studies have used separable convolutions as the tokenizer,^43,46^ a popular approach in EEG models since the ShallowConvNet and EEGNet CNNs.^29,47^ However, in our early experiments, we found this approach underperformed and was inefficient, leading us to discard it. In contrast to the original ViT model, which ‘patchified’ the input signal with a linear, non-overlapping tokenizer,^48^ we showed that a deep convolutional embedding results in higher performances. This improvement is likely due to the convolution’s inductive bias towards hierarchical dynamics across timescales and spatial scales.^49^ The discrepancies between our findings and previous studies on Transformer-based EEG models probably arise, in part, from dataset size and complexity: our training dataset included over 1 million samples from more than 900 patients, while prior studies used significantly smaller training samples (15 000–80 000 segments from 23–70 patients^43-45,50^) as well as shorter EEG segments (up to 50 000 points,^43-45,50^ compared to our 114 000 points per segment).

A notable advantage of Transformers over CNNs is their scalability. DeepEpilepsy showed continual improvement as the size of the training sample increased, without hitting a performance ceiling. Recent studies have further demonstrated CNNs’ limitations in scaling to large EEG datasets.^51^ While data augmentation through RandAugment increased model variability without clear performance benefits in our dataset, it might prove more valuable with larger training samples. The absence of a performance ceiling in DeepEpilepsy suggests potential for further improvements with larger datasets, motivating multicentre collaborations to expand the training sample.

Unlike other approaches to automated EEG interpretation that rely on explicit IED detection,^24,25,31,32^ DeepEpilepsy was trained without specific emphasis on IEDs. The model’s good performance in EEGs without IEDs (AUROC of 0.74) and its complementarity with IED-based classification suggests it captures additional epilepsy-specific patterns. Our embedding analysis suggests these patterns may be linked to changes in the higher frequency spectrum (40–100 Hz), which include the lower range of high-frequency oscillations (HFOs, typically 80–500 Hz). HFOs on intracranial EEG may have a prognostic value in patients with refractory temporal lobe epilepsies,^52,53^ and some studies have successfully detected them on scalp EEG with promising correlation with seizure outcomes.^54-56^ However, the role of HFOs on scalp EEG remains limited, largely due to technical challenges such as requirement for a high sampling frequency (most studies using >500 Hz) and low signal-to-noise ratio.^54^

The superiority of DeepEpilepsy over our benchmark model (LightGBM),^21^ which used carefully selected traditional EEG features (spectral power, nonlinear measures, peak alpha frequency), suggests that learning from raw EEG data captures relevant patterns that might be missed by conventional analysis. This is particularly evident in two aspects: the model’s sensitivity to high-frequency patterns (40–100 Hz) and its improved performance with longer segments (30 s), suggesting it captures both fine-scale spectral features and longer-term dynamics not typically considered in routine EEG interpretation. These findings warrant further investigation to better understand the clinical significance of these patterns.

Integrating DL models like DeepEpilepsy in the clinical workflow could enhance clinical decision-making by the increasing the information available in case of diagnostic uncertainty. However, this must be balanced against the risks of false positive predictions. While IEDs showed perfect specificity in our dataset, DeepEpilepsy’s improved sensitivity comes at the cost of lower specificity, which is particularly concerning given the significant impact of an incorrect epilepsy diagnosis (unnecessary medications, driving restrictions and psychosocial consequences).^57,58^

Therefore, we envision DeepEpilepsy as a decision support tool rather than a diagnostic test. A positive prediction by the model in a patient with neurological events of uncertain significance and negative workup (no IEDs on EEG, no epileptogenic lesion on MRI) could increase the suspicion of epilepsy, prompting to more frequent follow-ups or repeat EEGs. Conversely, a patient with a low pre-test probability of epilepsy, absence of IEDs and a negative DL prediction could reduce clinical suspicion. Most likely, combined with advances in other domains such as text processing, imaging and genetics,^59-61^ the automated EEG analysis will lead to a more comprehensive phenotyping of these patients and potentially lead to quantifying the seizure likelihood. This could also improve clinical trials in epilepsy, which are currently limited by self-reported and unreliable outcome measures.^15,62^

This study has limitations. Our data comes from a single centre, and although routine EEG recording is standardized, variability in hardware, software and technique may affect generalizability. Additionally, at our centre, patients with a first unprovoked seizure presenting at the emergency department generally undergo their EEG there and not as out-patient, limiting their inclusion in our cohort. Another limitation is the use of the EEG report as a measure of whether an EEG contains IEDs, which could be biased as EEG readers are not blinded to the diagnosis. However, for patients that were ‘undiagnosed’ at the time of the EEG, the limitation does not apply. Finally, subgroup analyses were limited by the relatively small sample size.

In conclusion, this study demonstrates that DeepEpilepsy, a Transformer model, could identify epilepsy on routine EEG independently of IEDs. The DL algorithm alone had an AUROC of 0.76, surpassing previously proposed methods, which was increased to 0.83 when combined with IEDs. Several questions remain such as the exact nature of brain dynamics captured by DeepEpilepsy, the optimal sample sizes for training the model and the true clinical impact of this increased diagnostic yield in specific clinical settings.

## Supplementary Material

fcaf319_Supplementary_Data

## Data Availability

The code for the study will be available upon publication at the following address: https://gitlab.com/chum-epilepsy/dl_epilepsy_reeg. Anonymized data will be made available to qualified investigators upon reasonable request, conditional to the approval by our REB. The STARD checklist is provided as Supplementary material.
