# Supplementary material for: Improving diagnostic accuracy of routine EEG for epilepsy using deep learning
Source: Brain Commun. 2025 Aug 25;7(5):fcaf319. doi: 10.1093/braincomms/fcaf319 (PMC12419360; doi:10.1093/braincomms/fcaf319)
Supplement: fcaf319_Supplementary_Data [file fcaf319_supplementary_data.zip › Supplementary_material.pdf]

Supplementary material

Supplementary Methods 1: Automated processing of EEG and classification

Deep Learning: We implemented two DL approaches. First, we adapted to EEG data the ConvNeXt model, a deep CNN analog to the ResNet, selected for its robust performance in computer vision tasks.[1] Second, we implemented a novel model coined *DeepEpilepsy*, based on the Vision Transformer (ViT) architecture, a Transformer model that takes images as input and that outputs class probabilities.[2] DeepEpilepsy uses a three-layer convolutional tokenizer plus a bottleneck convolution, restricting the complexity of the model and allowing to capture multi-scale features (**Figure 1**), akin to the Compact Convolutional Transformer.[3] We also tested a tokenizer with non-overlapping linear patch embedding proper to the original ViT model.[2]

For all DL models, EEGs were segmented into overlapping 10- or 30-second segments and scaled so that each channel had a mean of zero and standard error of one. These scaled segments were used as input into the DL models. To enhance model generalization, we applied a random data augmentation algorithm during training, similar to the RandAugm algorithm.[4] For each EEG segment, one augmentation was drawn randomly from a set of transformations, which included filtering (band-pass, low-pass, high-pass), masking (channel, time), and adding noise (**Supplementary Figure 1**). These augmentations were applied with a 50% probability. The intensity of the augmentation (e.g., filter frequency, noise level, mask length) was also randomized and controlled by a hyperparameter M. Based on initial experiments on the training and validation data, we set M = 8.

We performed a Bayesian hyperparameter search on the training and validation set to select four different configurations for the ViT (one of which was DeepEpilepsy) (**Supplementary Table 1**) and three for ConvNeXt (**Supplementary Table 2**). We also investigated different learning rates, weight decay, and batch size values. The final models were trained on the entire training and validation set. The optimization hyperparameters and model specifications are described in **Supplementary Table 4**.

ShallowConvNet: We reimplemented the ShallowConvNet model following the configuration outlined in Schirrmeister et al.[5] However, after conducting a hyperparameter search on the training and validation set, we identified a more optimal configuration specific to our dataset, which we used for testing (**Supplementary Table 3**). The EEG segmentation and standardization were consistent with the other DL models. Similarly, we optimized training hyperparameters (learning rates, weight decay, and batch size values) through a Bayesian search on the training and validation set (**Supplementary Table 4**).

EEG markers: We followed the methodology described in Lemoine et al.,[6] selecting only the best-performing markers and testing both 10- and 30-second segments. EEGs were segmented at pre-specified time points (every change of montage, every 15s during hyperventilation, every 15s for two minutes post-hyperventilation, every photic stimulation frequency, and every eye closure or opening). We applied an automated artifact detection/rejection algorithm (*AutoReject*)[7] and extracted the following markers: fuzzy entropy, line length, correlation dimension, band power, and peak alpha. Band power was calculated using a multitaper method, with integrals estimated using Simpson's method (frequency ranges: 100–75 Hz, 75–40 Hz, 40–20 Hz, 20–13 Hz, 13–10 Hz, 10–8 Hz, 8–6 Hz, 6–4 Hz, 4–2 Hz, and 2–1 Hz). For nonlinear features (fuzzy entropy, line length, and correlation dimension), the *Sym5* wavelet was used with six decomposition levels (with frequency ranges: 100–50 Hz, 50–25 Hz, 25–12.5 Hz, 12.5–6.25 Hz, 6.25–3.125 Hz, and 3.125–1.56 Hz).[8] One value was extracted per marker, EEG, segment, channel, and frequency band. Missing values were imputed using multivariate iterative imputation. Markers were used as input features for an L1-regularized boosted-trees classifier (LightGBM). The optimal hyperparameters for the classifier were selected via Bayesian optimisation using a 5-fold cross-validation on the training and validation set.

Supplementary Methods 2: Interpretability

We performed an exploratory analysis of the embeddings learned by DeepEpilepsy and ShallowConvNet to better understand which patterns were captured by these DL models from the raw EEG data. This analysis is distinct from the LightGBM benchmark model, which used pre-specified EEG features for classification.

For the embedding analysis, thirty-second segments from the testing set were processed through each DL model, and their embeddings (internal representation before the classification layer) were extracted. A clustering algorithm was then used to group the embeddings into 12 distinct clusters. To understand what patterns these clusters represented, we computed two traditional EEG features (band power and entropy) from the original EEG segments and analyzed how these features were distributed across the clusters. These features were computed using the same methods and frequency ranges described in the section **Automated processing of EEG and classification: EEG markers**.

To test for heterogeneity between clusters, we applied an analysis of variance (Krusper-Wallis test) at each frequency band. We then compared the F-score between both models and between frequency bands to identify which frequency ranges showed the greatest variation between clusters, suggesting these were important patterns learned by each model.

Supplementary Figure 1: Data augmentations used by the RandAugm algorithm

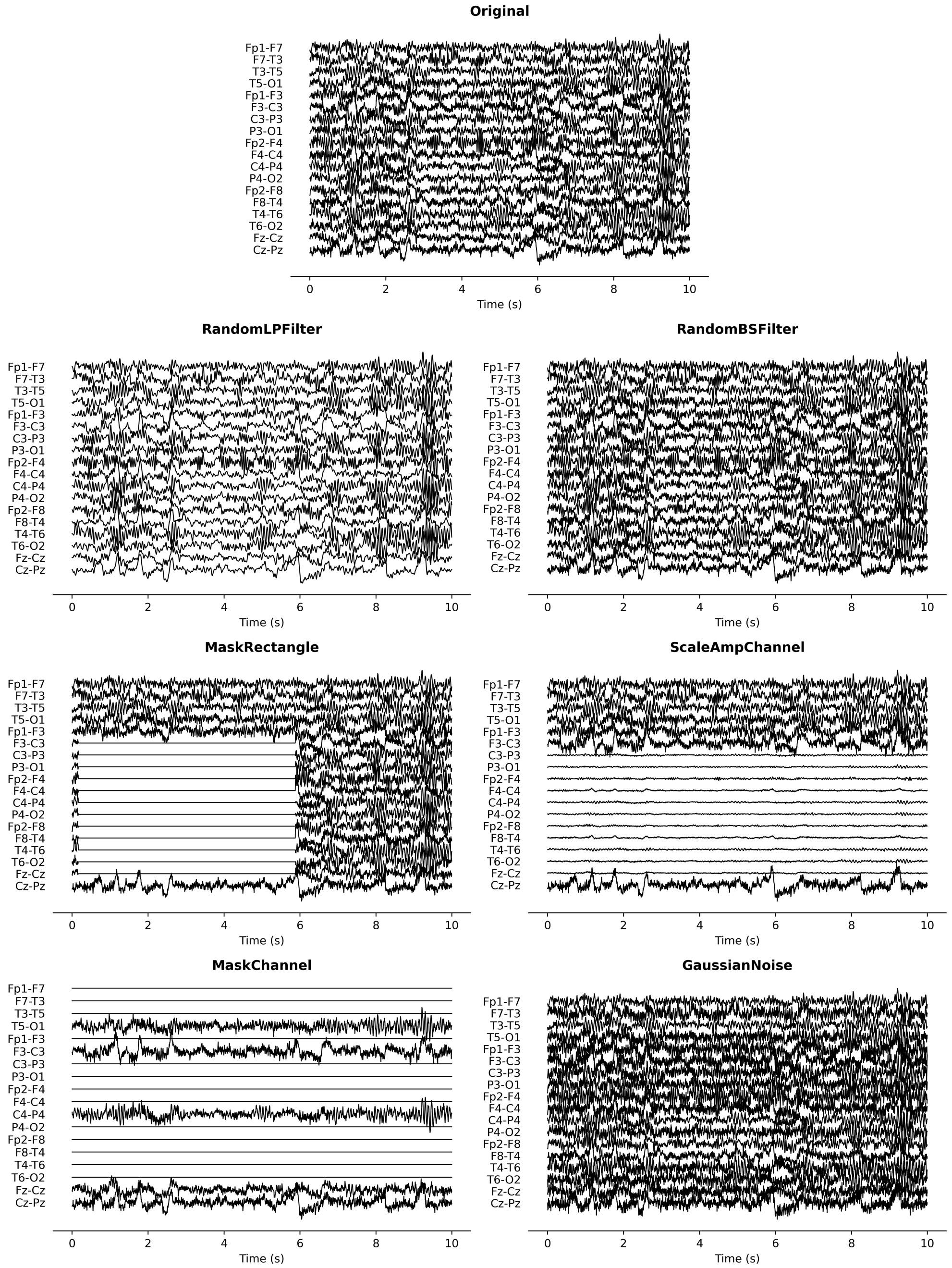

Supplementary Figure 1: Data augmentations used by the RandAugm algorithm, alongside the original EEG sample. RandomLPFilter: Low-pass filter, with random cut-off frequency. RandomBSFilter: band stop filter with randomly chosen frequency window. MaskRectangle: masking of data points contiguous in both time and space. ScaleAmpChannel: Random scaling of channels. MaskChannel: masking of all data points in randomly selected channels. GaussianNoise: Addition of gaussian noise with a random intensity. The intensity of the augmentations is scaled according to a hyperparameter M. For example, higher values of M result, on average, in lower values of cutoff frequencies for LPFilter, larger mask area for MaskRectangle, and a larger number of channels affected by ScaleAmpChannels as well as a higher amplitude of scaling.

Supplementary Table 1–4: Deep learning hyperparameters for the final model configurations

Supplementary Table 1: Model configurations for the Vision Transformer (ViT) models

Model	patch size	tokenizer	tokenizer: layers	hidden dim	layers	heads	MLP size	dropout	attention dropout	params (M)
ViT1d, linear, small	200	Linear	1	128	2	2	128	0.25	0.25	0.7
ViT1d, linear, large	50	Linear	1	512	6	8	512	0.25	0.25	10.0
ViT1d, Conv, small	200	Convolution	3	128	2	2	128	0.25	0.25	0.4
DeepEpilepsy: ViT1d, Conv, large	50	Convolution	3	512	6	8	512	0.25	0.25	10.6

Supplementary Table 2: Model configurations for the ConvNext models

Model	blocks	channels	stem: downsampling scale	drop path rate	params (M)
ConvNeXt, small	1, 1, 3, 1	16, 32, 64, 128	4	0.1	0.3
ConvNeXt, large	2, 2, 6, 2	32, 64, 128, 256	2	0.1	2.0
ConvNeXt, huge	3, 3, 9, 3	64, 128, 256, 512	2	0.1	11.9

Supplementary Table 3: Model configuration for the ShallowConvNet model

Model	kernel size (stride)	space conv channels	time conv channels	max pool window	dropout	params (M)
ShallowConvNet	16 (1)	64	128	80	0.25	0.040

Supplementary Table 4: Optimization parameters for all neural networks

Parameter	value
Optimizer	AdamW
Base learning rate	1.0e-5
Weight decay	0.05
Optimizer momentum	β1, β2=0.9, 0.999
Batch size	512
Training epochs	30
Learning rate schedule	Cosine decay
Warmup iterations	1000
Warmup schedule	Linear
RandAugm M	7
Gradient clipping	1.0 (ViT only)

Supplementary Table 5: Clinical characteristics of the "undiagnosed" subgroup of the testing cohort

	Epilepsy	No Epilepsy
Number of patients	28	47
Sex = woman (%)	19 (67.9)	26 (55.3)
Age (median [IQR])	41.00 [34.75, 58.25]	60.00 [50.50, 71.00]
Total follow-up after EEG in weeks (median [IQR])	119.00 [95.00, 134.50]	62.00 [17.00, 102.00]
Epilepsy type (%)		
Focal	23 (82.1)	–
Generalized	3 (10.7)	–
Unknown	2 (7.1)	–
Age of epilepsy onset (median [IQR])	37.00 [23.25, 52.00]	–
Seizure recurrence after EEG (%)	17 (60.7)	–
Number of days since last seizure (median [IQR])	87.50 [33.00, 164.00]	–
Number of epilepsy risk factors (median [IQR])	2.00 [1.00, 4.00]	1.00 [0.00, 3.00]
History of epilepsy surgery (%)	0 (0)	–
Number of ASM (%)		
0	9 (32.1)	42 (89.4)
1	12 (42.9)	5 (10.6)
2	5 (17.9)	0 (0.0)
3	2 (7.1)	0 (0.0)
4	0 (0.0)	0 (0.0)
5	0 (0.0)	0 (0.0)
Focal lesion on brain imaging (%)	10 (35.7)	10 (21.3)
Sleep deprived EEG (%)	9 (32.1)	8 (17.0)
IED (%)		
Absence	12 (42.9)	46 (97.9)
Presence	10 (35.7)	0 (0.0)
Uncertain	6 (21.4)	1 (2.1)
Abnormal slowing on EEG (%)	10 (35.7)	10 (21.3)

Supplementary Figure 2: Performance of DeepEpilepsy compared to interictal epileptiform discharges on the undiagnosed subgroup.

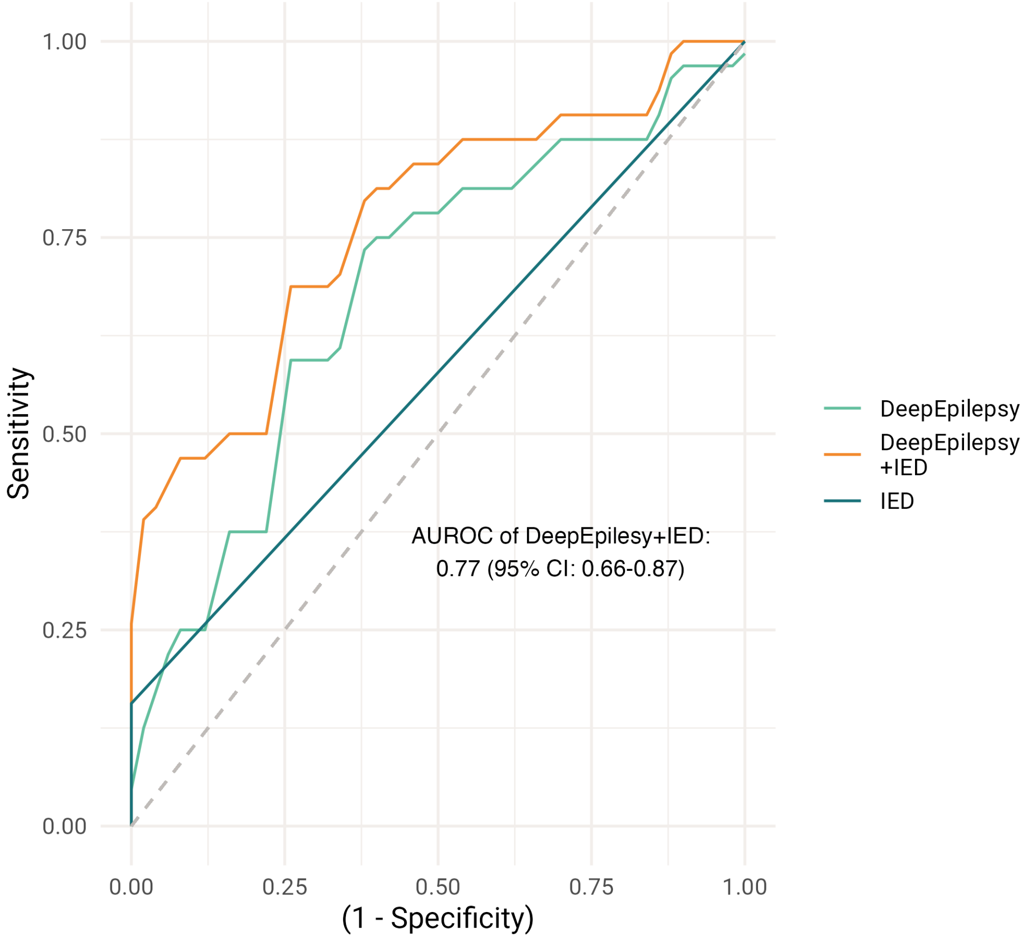

Supplementary Figure 2: ROC curves for IEDs only, DeepEpilepsy, and DeepEpilepsy combined with IEDs in the subgroup of patients not diagnosed with epilepsy at the time of the EEG (*n* = 77). AUROC: Area under the receiver operating characteristic curve; IED: interictal epileptiform discharges.

Segment duration and RandAugment Analysis

Supplementary Table 6: Effect of segment duration on DeepEpilepsy's performances

Segment duration (s)	AUROC (95% CI): RandAugment	AUROC (95% CI): No RandAugment
5	0.721 (0.639–0.804)	0.711 (0.625–0.792)
10	0.713 (0.632–0.789)	0.691 (0.603–0.772)
**30**	**0.746 (0.663–0.821)**	**0.679 (0.589–0.763)**
45	0.733 (0.644–0.815)	0.717 (0.627–0.798)
60	0.716 (0.634–0.796)	0.705 (0.615–0.787)

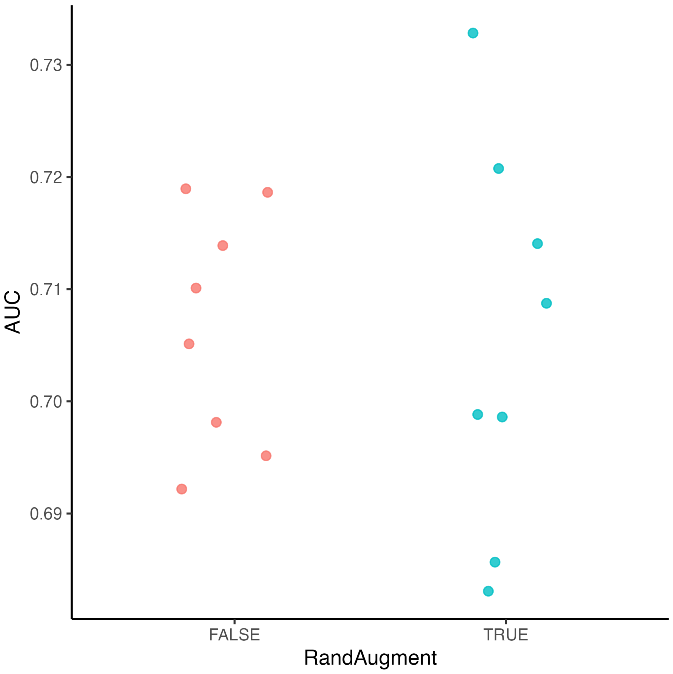

Supplementary Figure 3: Effect of RandAugment on DeepEpilepsy's performances. Each point represents the AUROC achieved by DeepEpilepsy on the testing set after independent training runs (20 epochs each) with (blue) or without (orange) RandAugment data augmentation. AUC: area under the curve.

Supplementary Figure 4: Power spectrum density of EEG segments clustered according to their latent representations using DeepEpilepsy vs. ShallowConvNet

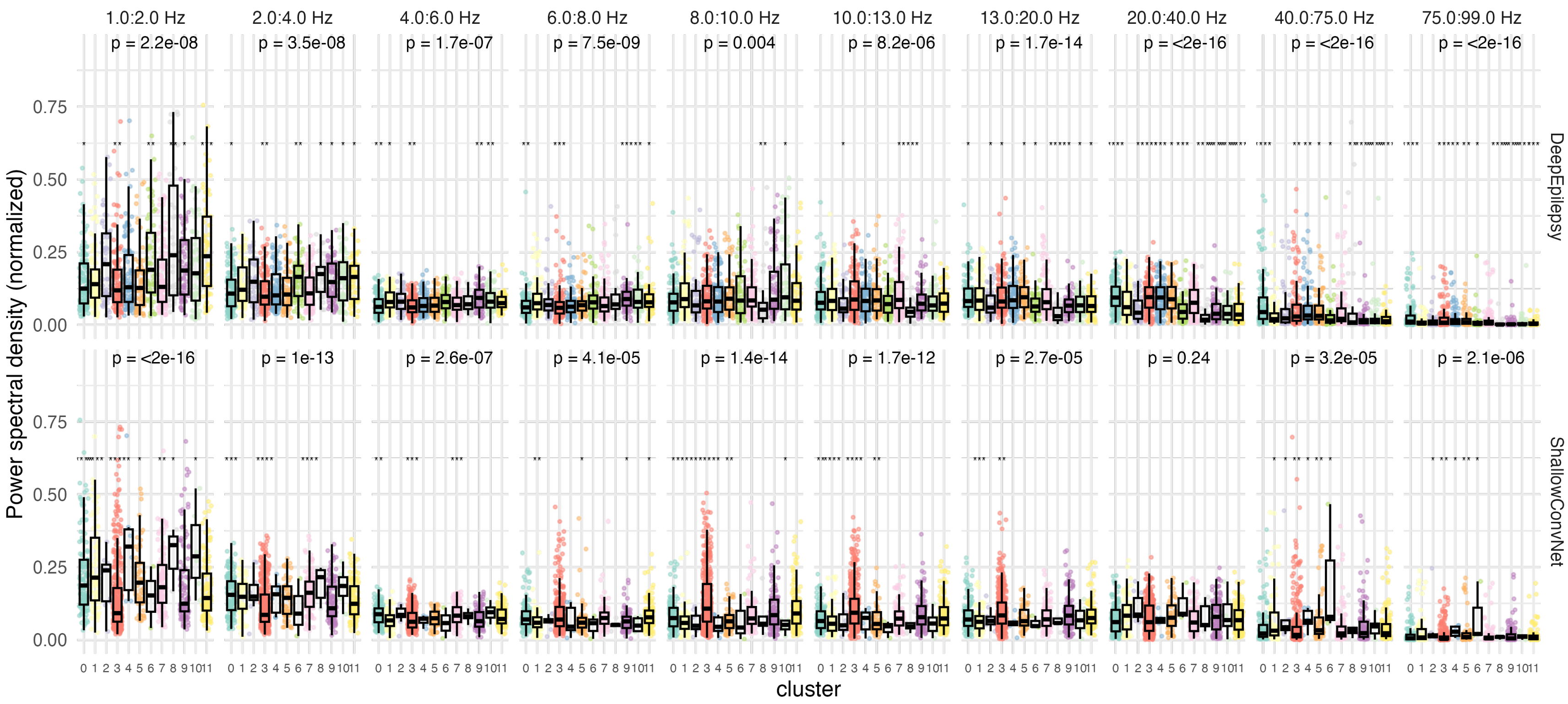

Supplementary Figure 4: Power spectrum density of 30s EEG segments clustered according to their latent representations using DeepEpilepsy vs. ShallowConvNet. Each point represent the normalized power spectrum density for individual EEG segments of 30s at different frequency band for both models. Each EEG segments was processed through either the trained ShallowConvNet (bottom) or the trained DeepEpilepsy (top) to generate a latent vector. The latent vectors were then clustered using K-means clustering (K=12). The power in each band was calculated for the input segment (1 Hz:2 Hz, 2 Hz:4 Hz, etc.) and plotted on the y-axis. A statistical analysis of inter-cluster variance was perform in each frequency band using the Krusper-Wallis test (*p*-values at the top of each facet, *n* = 1 024 segment per test). A lower p-value correspond to a larger heterogeneity between clusters in that frequency bands.

Supplementary Figure 5: Entropy of EEG segments clustered according to their latent representations using DeepEpilepsy vs. ShallowConvNet

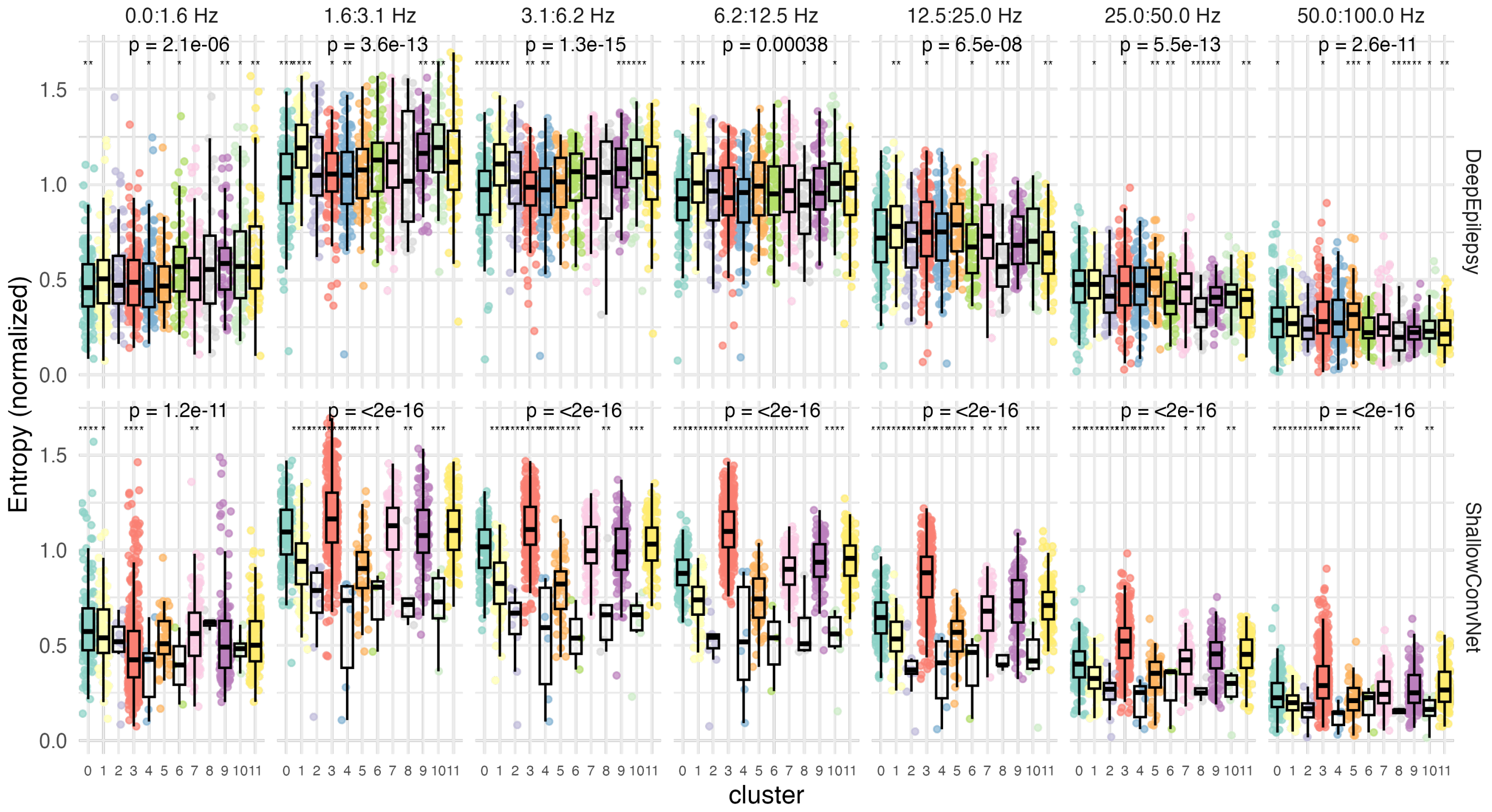

Supplementary Figure 5: Entropy of EEG segments clustered according to their latent representations using DeepEpilepsy vs. ShallowConvNet. Each point represent the normalized entropy for individual EEG segments of 30s at different frequency band for both models. Each EEG segments was processed through either the trained ShallowConvNet (bottom) or the trained DeepEpilepsy (top) to generate a latent vector. The latent vectors were then clustered using K-means clustering (K=12). The entropy in each band was calculated for the input segment (1 Hz:2 Hz, 2 Hz:4 Hz, etc.) and plotted on the y-axis. The fuzzy entropy algorithm was used with parameters m=2 and r=0.2. A statistical analysis of inter-cluster variance was perform in each frequency band using the Krusper-Wallis test (p-values at the top of each facet, n = 1 024 segment per test). A lower p-value correspond to a larger heterogeneity between clusters in that frequency bands.

References

1. Liu Z, Mao H, Wu CY, Feichtenhofer C, Darrell T, Xie S. A ConvNet for the 2020s. *arXiv:220103545 [cs]*. Published online March 2, 2022. Accessed March 27, 2022. http://arxiv.org/abs/2201.03545

2. Dosovitskiy A, Beyer L, Kolesnikov A, et al. An Image is Worth 16x16 Words: Transformers for Image Recognition at Scale. *arXiv:201011929 [cs]*. Published online June 3, 2021. Accessed January 29, 2022. http://arxiv.org/abs/2010.11929

3. Hassani A, Walton S, Shah N, Abuduweili A, Li J, Shi H. Escaping the Big Data Paradigm with Compact Transformers. Published online June 7, 2022. Accessed September 30, 2023. http://arxiv.org/abs/2104.05704

4. Cubuk ED, Zoph B, Shlens J, Le QV. RandAugment: Practical automated data augmentation with a reduced search space. Published online November 13, 2019. doi:10.48550/arXiv.1909.13719

5. R. Schirrmeister, L. Gemein, K. Eggensperger, F. Hutter, T. Ball. Deep learning with convolutional neural networks for decoding and visualization of EEG pathology. In: *2017 IEEE Signal Processing in Medicine and Biology Symposium (SPMB)*. ; 2017:1-7. doi:10.1109/SPMB.2017.8257015

6. Lemoine É, Toffa D, Pelletier-Mc Duff G, et al. Machine-learning for the prediction of one-year seizure recurrence based on routine electroencephalography. *Scientific Reports*. 2023;13(1):12650. doi:10.1038/s41598-023-39799-8

7. Jas M, Engemann DA, Bekhti Y, Raimondo F, Gramfort A. Autoreject: Automated artifact rejection for MEG and EEG data. *NeuroImage*. 2017;159:417-429. doi:10.1016/j.neuroimage.2017.06.030

8. Gandhi T, Panigrahi BK, Anand S. A comparative study of wavelet families for EEG signal classification. *Neurocomputing*. 2011;74(17):3051-3057. doi:10.1016/j.neucom.2011.04.029
