# Supplementary figures and images for: Improving diagnostic accuracy of routine EEG for epilepsy using deep learning
Source: Brain Commun. 2025 Aug 25;7(5):fcaf319. doi: 10.1093/braincomms/fcaf319 (PMC12419360; doi:10.1093/braincomms/fcaf319)

**A**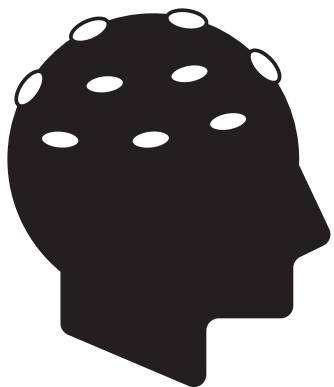

20-60 min routine EEG

**B**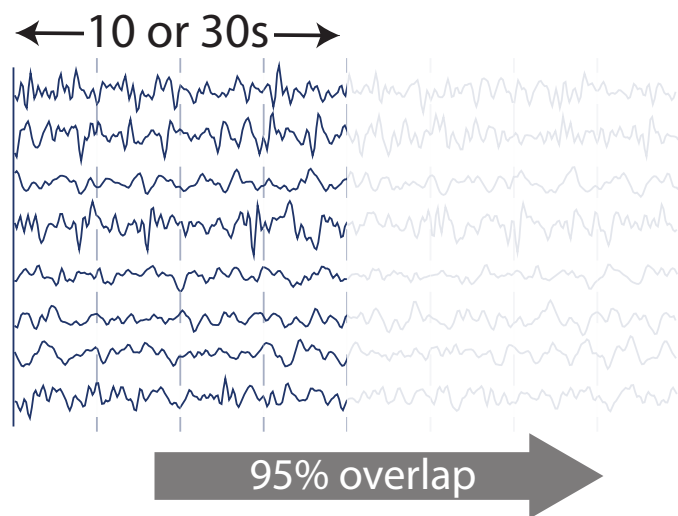**C**

RandAugment

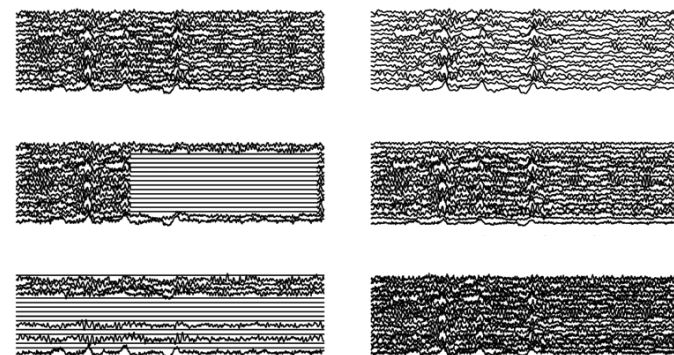**D**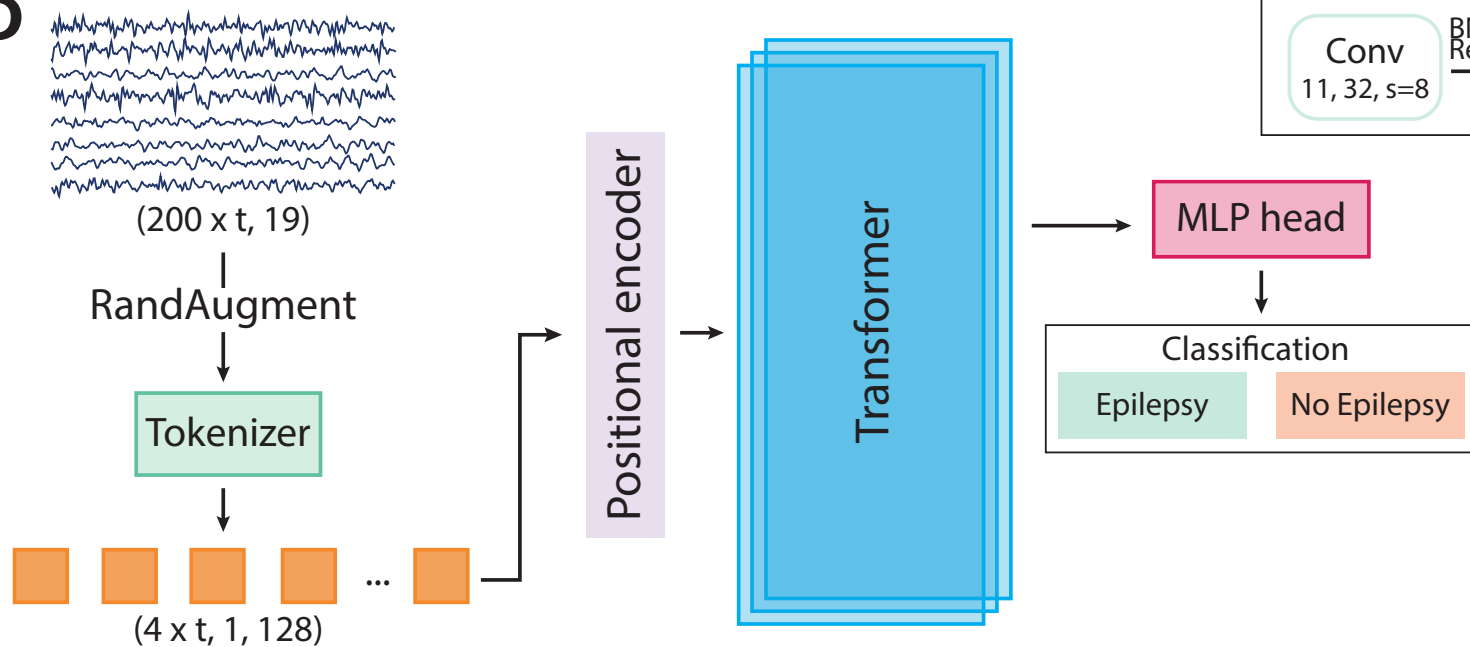**Tokenizer**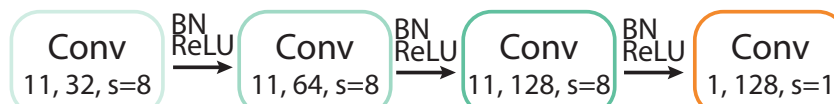**Transformer block**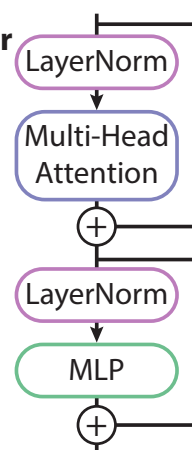

Supplement: fcaf319_Supplementary_Data [file fcaf319_supplementary_data.zip › Figure 1 A4.pdf]
